# SynGAP Regulates Protein Synthesis and Homeostatic Synaptic Plasticity in Developing Cortical Networks

**DOI:** 10.1371/journal.pone.0083941

**Published:** 2013-12-31

**Authors:** Chih-Chieh Wang, Richard G. Held, Benjamin J. Hall

**Affiliations:** 1 Neuroscience Program, School of Science and Engineering, Tulane University, New Orleans, Louisiana, United States of America; 2 Department of Cell and Molecular Biology, School of Science and Engineering, Tulane University, New Orleans, Louisiana, United States of America; Institute for Interdisciplinary Neuroscience, France

## Abstract

Disrupting the balance between excitatory and inhibitory neurotransmission in the developing brain has been causally linked with intellectual disability (ID) and autism spectrum disorders (ASD). Excitatory synapse strength is regulated in the central nervous system by controlling the number of postsynaptic α-amino-3-hydroxy-5-methyl-4-isoxazolepropionic acid receptors (AMPARs). *De novo* genetic mutations of the synaptic GTPase-activating protein (SynGAP) are associated with ID and ASD. SynGAP is enriched at excitatory synapses and genetic suppression of SynGAP increases excitatory synaptic strength. However, exactly how SynGAP acts to maintain synaptic AMPAR content is unclear. We show here that SynGAP limits excitatory synaptic strength, in part, by suppressing protein synthesis in cortical neurons. The data presented here from *in vitro*, rat and mouse cortical networks, demonstrate that regulation of translation by SynGAP involves ERK, mTOR, and the small GTP-binding protein Rheb. Furthermore, these data show that GluN2B-containing NMDARs and the cognitive kinase CaMKII act upstream of SynGAP and that this signaling cascade is required for proper translation-dependent homeostatic synaptic plasticity of excitatory synapses in developing cortical networks.

## Introduction

Neurodevelopmental disorders such as non-syndromic forms of ID and ASD involve disruptions in excitatory synaptic function. ASDs affect 1% of the general population and are characterized by deficits in social interactions, communication, and manifestation of repetitive behaviors [1]. Dysregulated neurotransmission such as enhanced ratio of excitatory/inhibitory (E/I) synaptic balance has been proposed to underlie ASD [2]. Supporting this hypothesis, abnormal increases in synaptic strength have been demonstrated in mouse models of ASD [3]–[6]. Fast excitatory synaptic transmission in the brain is mediated by the alpha-amino-3-hydroxy-5-methyl-4-isoxazole propionic acid receptors (AMPARs) and N-methyl D-aspartate receptors (NMDARs). Basal levels of AMPARs are tightly regulated allowing neurons to adjust synaptic strength rapidly in response to changes in network activity.

Synaptic GTPase activating protein (SynGAP), one of the most abundant postsynaptic density (PSD) proteins, is associated with NMDAR complexes and is activated by CaMKII [7]–[9]. *De novo* mutations in SynGAP have been identified in patients with ASD [10]–[12]. Moreover, heterozygous SynGAP mice display autistic-like behaviors and cognitive deficits [13], [14]. These altered behaviors are likely due to imbalance in synaptic E/I ratio, since deletion of SynGAP increases dendritic spine size and enhances AMPAR-mediated synaptic currents during a critical period of circuit formation [14]–[16]. However, our understanding of how SynGAP regulates AMPAR content at developing excitatory synapses is incomplete.

SynGAP physically associates with NMDAR complexes via the PSD associated proteins SAP102 and PSD95 [7]. The NMDAR is a tetrameric protein complex composed of two obligatory GluN1 subunits and two GluN2 subunits. GluN2 subunits are encoded by four different genes, GluN2 A–D. GluN2A and GluN2B are the dominant subunits at cortical synapses with GluN2B expressed exclusively at immature synapses, whereas expression of GluN2A increases during synapse maturation. Ca^2+^ passing through NMDARs can activate CaMKII, which preferentially interacts with GluN2B over GluN2A [17]–[20]. GluN2B-containing NMDARs are required for proper homeostatic synaptic plasticity (HSP) [21]. HSP is critical for maintaining neuronal activity within an appropriate range in response to changes in the integrated synaptic input a neuron receives and HSP can be regulated transcriptionally or translationally [22]–[27]. Although several molecules have been identified that regulate HSP the molecular mechanisms remain incompletely understood and a role for SynGAP has not been demonstrated.

In this study we demonstrate that SynGAP limits synaptic AMPAR content by suppressing protein synthesis. Removal of SynGAP from individual neurons in young cortical networks results in enhanced protein synthesis rates via signaling pathways involving ERK, mTOR, and Ras homolog enriched in brain (Rheb) and an increase in the synaptic contribution of GluR2-lacking AMPARs. Furthermore, we show translation-dependent, but not transcription-dependent, HSP is impaired in cortical neurons expressing siRNA targeted against SynGAP. We also provide evidence that NMDARs containing the GluN2B subunit and CaMKII are upstream effectors of SynGAP signaling. Our data provide a molecular basis to explain how SynGAP regulates synaptic strength, while adding to our understanding of NMDAR-mediated signaling and providing an important link in our understanding of brain disorders such as ASD caused by SynGAP dysfunction.

## Materials and Methods

### Ethics Statement

All animal use followed NIH guidelines and was in compliance with Tulane University IACUC Committee policy and procedures, animal welfare assurance number A4499-01. All experiments in this study are approved under the current (Oct. 2011–Oct. 2014) Tulane University IACUC committee protocols #0364 and 0363 issued to the PI.

### Cell Culture

Cell cultures were prepared as previously described [28]. All neuronal cultures used for electrophysiology recordings were plated at a concentration of 1×10^6^ cells/mL. For immunostaining and FUNCAT experiments low-density cultures were used that were either 0.25×10^6^ cells/mL for rat-derived cultures or 0.35×10^6^ cells/mL for mouse cultures. GluN2BKO and 2B->2A mouse cortical cultures were generated from E16–E17 embryos derived from heterozygous GluN2B or 2B->2A matings in order to generate both WT and homozygous mutant sister cultures [21], [29]. Sex of individual embryos was not determined and therefore all the data is from both male and female brains.

### Transfection

Cultures were transfected on DIV 7 or 8 using Lipofectamine 2000 (Invitrogen) in Opti-MEM media (Invitrogen) or neurobasal medium with glutamate and 25 µM β-mercaptoethanol. SynGAPsiRNA was generated in p-Silencer vector (Invitrogen) using previously described and characterized target sequences recognizing the alpha isoform of SynGAP [30]. SynGAPsiRNA targeted the 19 bp sequence CCAGCAAGATCCTGATGCA while the scrambled control sequence was GCGACATAGCGCACATACT. WT SynGAP cDNA was provided by G. Rumbaugh (Scripps Research Institute, Florida). CaMKII and CaMKII-T286D cDNAs were courtesy of G. Patrick (University of California, San Diego). mTOR and Rheb cDNAs were purchased from Addgene (Cambridge, MA). All data were recorded between 11 and 16 DIV. For all siRNA based experiments data were generated at least 3, but not more than 6, days post-transfection (i.e., max 14 DIV for 8 DIV transfection). Plasmids were transfected at a ratio of 5∶1 relative to GFP in order to maximize co-expression in GFP positive cells. In preliminary experiments we found that mEPSC amplitudes in SynGAP siRNA expressing cells were significantly increased after two days post-transfection. The intensity of anti-SynGAP staining in siRNA expressing cells at four days post-transfection was 42.8% (±4.8) of controls cells while co-expression of WT SynGAP recovered this to 77.9% (±6.63) of control cells. Neither amplitude nor frequency (IEI) of mEPSCs was altered in GFP-alone expressing neurons, compared to non-transfected control cells: non-transfected (n = 24) = 12.68 pA vs. GFP (n = 17) = 12.96 (p = 0.67) and 4.57 s vs. 3.89 (p = 0.57).

### Electrophysiology

Synaptic activity was recorded in cell cultures while perfused at room temperature in a bicarbonate buffered solution containing 124 mM NaCl, 5 mM KCl, 26 mM NaHCO_3_, 1.23 mM NaH_2_PO_4_, 1.5 mM MgCl_2_, 2 mM CaCl_2_, and 10 mM glucose and bubbled constantly with 95% O_2_/5% CO_2_. Whole-cell voltage-clamp recordings were made using glass microelectrodes (borosilicate glass 1.5 mm outer diameter and 0.86 mm inner diameter, Warner Instruments, Hamden, CT), pulled on a micropipette puller (Flaming-Brown P-80/PC, Sutter Instrument, Novato, CA), and filled with a cesium-based intracellular solution containing 10 mM CsCl, 105 mM CsMeSO3, 8 mM NaCl, 0.5 mM ATP, 0.3 mM GTP, 10 mM HEPES, 5 mM glucose, 2 mM MgCl2, and 1 mM EGTA (pH 7.3). Pipette resistances ranged from 3–8 MΩ. Series access resistance ranged from 7 to 17 MΩ and was monitored for consistency. Signals were recorded using a patch-clamp amplifier (PC505B, Warner Instruments) and digitized with a BNC-2111 A/D board (National Instruments, Austin, TX) using custom-written software in Igor Pro (WaveMetrics, Lake Oswego, OR). Signals were amplified, sampled at 10 kHz, filtered to 2 or 5 kHz, and analyzed using custom routines in Igor Pro. Only cells with more than 5 events during the 100 s recording session were analyzed, mEPSC amplitudes ranged from 3–100 pA. Transfected cells were identified under epifluorescent visualization and then patched under DIC optics on an Olympus BX-51 upright microscope. Only cells with leak current values less then 250 pA after breakthrough and consistent over time were used for recording. TTX, APV, picrotoxin, NASPM, actinomycin-D, anisomycin, KN93, rapamycin, PKI 14–22, and KU-0063794 were all acquired from Tocris Bioscience (Ellisville, MO). U0126, PMA and an inactive version of PMA, P-2170 were from LC Laboratories (Woburn, BA). All recordings were performed on pyramidal neurons, ‘n’ represents cell number and all recordings were done in multiple cultures.

### Immunohistochemistry

Cells were fixed with 4% sucrose-containing PFA for 30 min then incubated in blocking solution (PBS with 0.1% Triton-X and 3% Donkey Serum) for 1 hour. For parallel comparisons cells were immunostained and imaged on the same day. Primary antibodies were added in blocking solution for 3 hours at room temperature or overnight at 4°C. Secondary antibodies were added in the blocking solution for 1 hour at room temperature. The following primary antibodies were used at 1∶1000 dilution: anti-MAP2 (Abcam), anti-GFP (NeuroMab), anti-Rheb (NewEast Biosciences), anti-Rheb-GTP (NewEast Biosciences), anti-P70S6K and anti-phospho-P70S6K (T-389)(Cell Signaling Technologies). Images for primary, secondary, or tertiary dendrites proximal to the cell body were selected randomly for analysis. Image J was used for analysis. For all immunofluorescence-based data ‘n’ represents the number of individual cells, combined data were generated from multiple cultures.

### Visualization of Newly Synthesized Proteins Using FUNCAT

Fluorescent non-canonical amino acid tagging (FUNCAT) was used to visualize protein synthesis as per the methods in [31]. The azide (L-azidohomoalanine; AHA) tag (cat# C10102), reaction buffer kit (cat# C10269), and alkyne-containing click chemistry reagents (cat# A10275) were all purchased from Invitrogen. Cells were incubated in methionine-free DMEM medium (Invitrogen) for 1 hour prior to AHA exposure in order to deplete endogenous methionine. Cells were then incubated with methionine-free medium containing 50 µM AHA for 6 hours followed with fixation for 30 min and permeabilization for 30 min. Cells were then incubated with the click-iT reaction cocktail for 1 hour. For all FUNCAT data ‘n’ represents individual cells and combined data were generated from multiple cultures.

### Statistical Analysis

**Figure 1:**
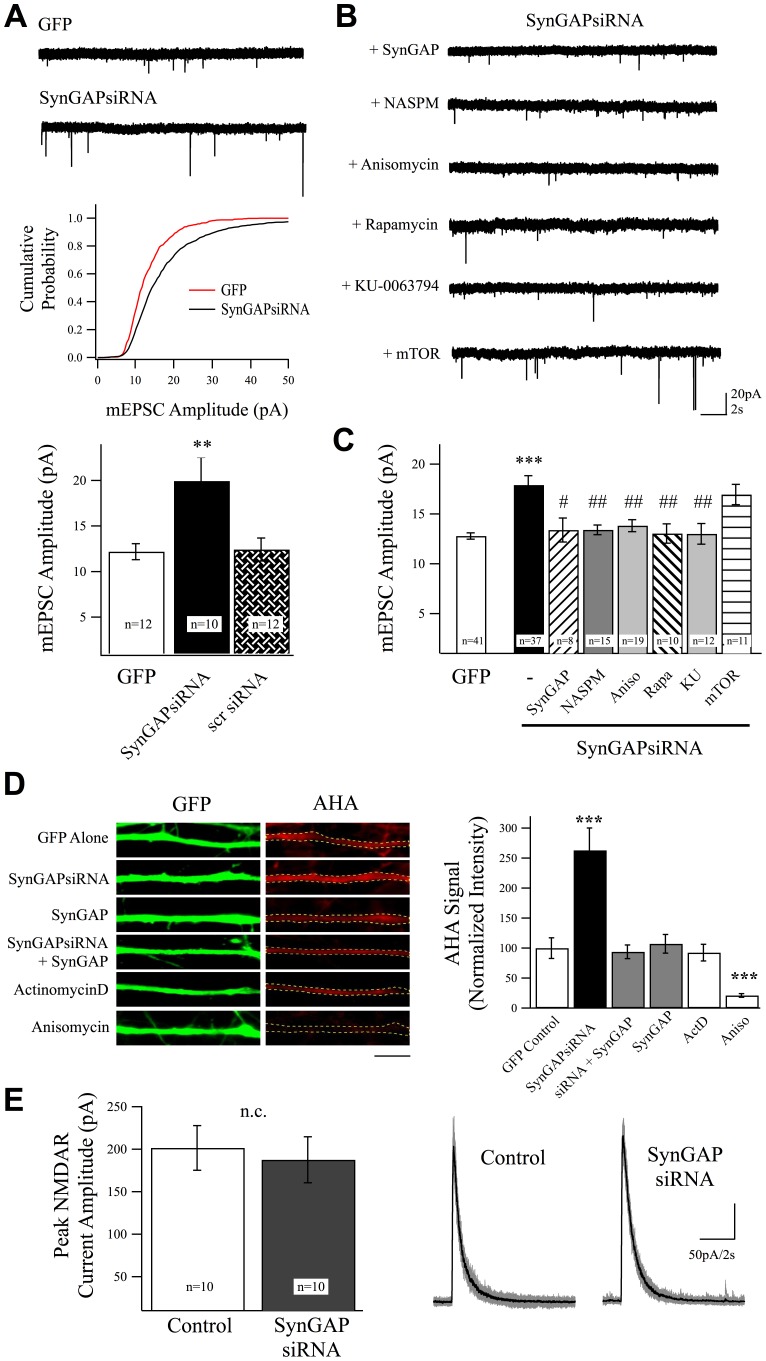
Removal of SynGAP Increases Synaptic AMPARs in a Protein Synthesis and mTOR Dependent Manner. (A) Example traces of whole-cell current recordings clamped at −65 mV from cortical neurons transfected with GFP or GFP+ siRNA directed against SynGAP (SynGAPsiRNA). Middle: cumulative histogram showing the distribution of mEPSC amplitudes in each experimental condition, the increase in SynGAPsiRNA event sizes was highly significant as determined by K-S nonparametric test. Lower: increased mEPSC amplitudes were significantly increased by expression of SynGAP siRNA but not a scrambled control (scr) siRNA. (B) Co-expression of SynGAP recovered AMPAR levels in SynGAPsiRNA expressing neurons. The increase in mean mEPSC amplitude was also recovered by treatment with the GluR2-lacking AMPAR antagonist NASPM (10 µM), treatment with the protein synthesis blocker anisomycin (Aniso, 40 µM, 6 hr), or mTOR blockers rapamycin (Rapa, 1 µM, 6 hr) or KU-0063794 (1 µM, 5 hr) also suppressed the increase in mean mEPSC amplitudes. mTOR overexpression increased mEPSC amplitudes in control cells, but did not cause a further increase in cells expressing SynGAPsiRNA. (C) Mean values (±SEM) and results from statistical analysis by Dunnett's multiple comparison test are shown for all experimental conditions. (D) Representative images and normalized mean (±SEM) AHA signal values from experiments visualizing new protein synthesis using FUNCAT. Knockdown of SynGAP significantly increased rates of protein synthesis as measured by rates of AHA incorporation, while co-expression of WT SynGAP recovered protein synthesis rates. AHA incorporation, as predicted, was sensitive to the protein synthesis blocker anisomycin (aniso) but not the transcription inhibitor actinomycin-D (act-D). Scale bar  = 5 µm. (E) NMDAR currents evoked by local NMDA + d-serine application were unchanged in SynGAP siRNA expressing cells compared to control GFP expressing neurons.

Statistical significance was determined using Dunnett's test for multiple comparisons on mean values obtained from each genotype (or experimental condition) and compared to controls. Individual control neurons from sister cultures and GFP transfections were generated for the conditions tested. For mEPSC recordings GFP and non-transfected neurons showed no differences and are grouped as controls. All data (text and figures) are presented as mean ±SEM. */# p<0.05, **/## p<0.01, and ***/### p<0.001. For statistical analysis of the mEPSC distributions in Figure 1A a non-parametric Kolmogorov-Smirnov test was used.

## Results

### SynGAP Regulates Excitatory Synaptic Strength by Suppressing AMPAR Content

Global genetic knockout of SynGAP results in increased excitatory synaptic function in mice [15], [16], [32]. Consistent with this, overexpression of SynGAP in cultured neurons suppresses AMPAR content at hippocampal synapses, in an isoform specific manner [32]. We found here that knocking down expression of alpha SynGAP, by single-cell siRNA transfection in cortical neurons, significantly increased mEPSC amplitudes relative to control cells (Fig. 1A). Neurons were transfected using Lipofectamine 2000 and AMPAR content at developing cortical synapses was determined by isolating AMPAR-mediated miniature excitatory postsynaptic currents (mEPSCs) in cortical cultures between 11–16 days in vitro (DIV) (see methods for details). Individual neurons were voltage-clamped at −65 mV while perfusing with 0.5 µM tetrodotoxin (TTX) and 50 µM picrotoxin. For these experiments, cells were co-transfected with GFP as a cell marker and experimental conditions were compared to littermate control neurons generated and maintained in parallel. The frequency of events was not consistently altered in SynGAP siRNA expressing cells under these experimental conditions; mean inter-event interval for GFP controls was 4.29±0.57 s (n = 41) vs. 2.85±0.25 s (n = 37) for SynGAP siRNA. This trend to decrease in inter-event interval could be secondary to the increased amplitude of events reflecting an increase in the number of events above threshold for recording; however, it could also reflect changes in presynaptic release properties. While this second explanation would suggest a retrograde signaling mechanism due to the low proportion of transfected cells in these cultures, we did not look into it further as the frequency change was inconsistent and non-significant over the entire experimental group (n = 37) and (n = 41) neurons. We therefore focused on the mechanisms underlying the post-synaptic change in AMPAR-mediated current in response to SynGAP loss of function.

We found that the increase in mEPSC amplitudes in SynGAP knockdown cells could be rescued by co-expression of WT SynGAP and was due to enhanced incorporation of GluR2-lacking AMPARs, because treatment with NASPM (a polyamine toxin that specifically blocks AMPARs lacking the GluR2 subunit; 10 µM; cultures were pretreated with NSAPM for 10 min and continually bathed in NASPM during recording) recovered the siRNA mediated increase in mEPSC amplitudes to controls levels (Fig. 1B&C). Thus, we concluded that at this age in culture SynGAP strongly restricts insertion of GluR2-lacking AMPARs at individual excitatory synapses. In this way it may act as a break to prevent over strengthening of these synapses during development and would limit Ca^2+^ permeability of the synaptic AMPAR pool. Interestingly, the general protein synthesis inhibitor anisomycin also rescued the increase in amplitude in synGAPsiRNA expressing neurons suggesting that SynGAP regulates protein synthesis (Fig. 1B&C).

Mammalian target of rapamycin (mTOR) is a critical factor driving cell growth, proliferation, and survival, and has been implicated in a variety of diseases including ASD [33]–[35]. Activation of mTOR signaling leads to an increase in protein synthesis that can result in enhanced synaptic transmission [36], [37]. In light of this, we investigated whether or not mTOR is a downstream effector of SynGAP by using the mTOR specific antagonist rapamycin. As shown in figure 1, pretreatment with rapamycin (1 µM, 6 hr) recovered mEPSC amplitudes to control levels in SynGAPsiRNA expressing neurons. To confirm this we also showed that pretreatment with another mTOR antagonist KU-0063794 (1 µM) is effective in recovering mean mEPSC amplitudes to control levels (Fig. 1B&C). If mTOR is indeed a critical mediator downstream of SynGAP to control synaptic AMPAR content, we predicted overexpression of mTOR should enhance AMPAR incorporation, and that this effect would be occluded in SynGAPsiRNA expressing neurons. As predicted, neurons transfected with mTOR displayed increased mEPSC amplitudes compared to GFP transfected control neurons (mTOR o.e.: 15.8±1.2 pA, n = 14 vs. GFP: 12.4±0.6 pA, n = 41; p<0.001), however any additional increase of AMPAR-mEPSC amplitudes was occluded in SynGAPsiRNA expressing neurons (Fig. 1B&C). These data suggest SynGAP regulates AMPAR content at individual synapses in a protein synthesis- and mTOR-dependent manner.

### SynGAP Regulates mTOR to Control Excitatory Synaptic Strength

Synaptic AMPAR content can be regulated at the level of DNA transcription, mRNA translation, protein trafficking, and protein degradation. Previous studies reported that SynGAP regulates AMPAR synaptic incorporation by controlling AMPAR trafficking [16]. However, the exact details of this regulation remain unclear. In light of the data in figure 1, as well as the fact that the translation machinery is dysregulated in several ASD mouse models [5], [34], [35], [38] and SynGAP mutant mice display autistic-like behaviors, we wanted to directly measure protein synthesis rates under these different experimental conditions. To accomplish this, we used FUNCAT (fluorescent non-canonical amino acid tagging) [31]. In this technique l-azidohomoalanine (AHA), which mimics methionine, is applied to label newly synthesized proteins. Endogenous methionine was depleted for 1 hour before AHA application. We first determined that AHA-labeling measures rates of translation specifically by showing that only the translation blocker anisomycin, but not the transcription blocker actinomycin-D, suppressed AHA incorporation (Fig. 1D). As expected, single-cell knockdown of SynGAP using siRNA expression significantly increased AHA signal in dendrites, showing that SynGAP normally suppresses protein synthesis (Fig. 1D). Interestingly, while SynGAP co-expression was able to recover the increase in AHA signal seen in siRNA expressing neurons back to control levels, overexpression of SynGAP alone did not suppress protein synthesis rates compared to control neurons (Fig. 1D). This suggests that endogenous SynGAP strongly suppresses protein synthesis under basal, non-stimulated, conditions. In order to assess the specificity of this regulation we also examined NMDAR levels by measuring the current evoked by localized NMDA + d-serine application in control GFP transfected and SynGAP siRNA expressing neurons. We saw no change in NMDA-evoked currents after knockdown of SynGAP (Fig. 1E). We also surface stained these neurons using an antibody against GluN1 and saw no change in levels of this obligatory NMDAR subunit between GFP control neurons and siRNA expressing neurons: GFP 100±6.03 (n = 21); SynGAPsiRNA 96.1±8.14; (n = 22); p = 0.7.

### SynGAP Regulates ERK Signaling to Control Synaptic Strength

**Figure 2:**
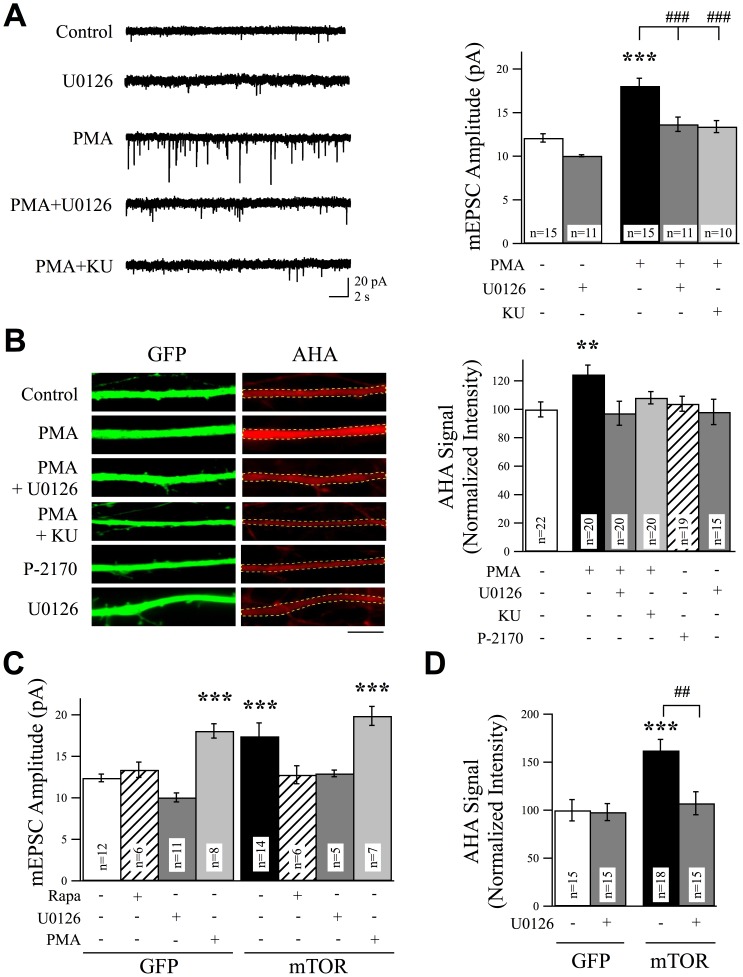
ERK Positively Regulates Synaptic AMPAR Content by Increasing Protein Synthesis via mTOR. (A) Representative mEPSC traces and combined data from cells in the presence or absence of the ERK blocker U0126 (25 µM, 6 hr) or ERK activator phorbol 12-myristate 13-acetate (PMA; 1 µg/ml, 1 hr). ERK blockade suppressed mEPSC amplitudes, whereas ERK activation increased mEPSC amplitudes in a manner that could be suppressed by U0126 or the mTOR antagonist KU-0063794 (KU). For co-treatment experiments cells were preincubated with U0126 or KU-0063794 before PMA application. (B) Example images and quantification from FUNCAT experiments of cortical neurons treated with U0126, PMA, or an inactive PMA analog, P-2170. Scale bar  = 5 µm. PMA-induced ERK activation resulted in a significant increase in synthesis of dendritic protein. (C) ERK regulates synaptic AMPAR content in an mTOR-dependent manner. Overexpression of mTOR produced a significant increase in mEPSC amplitudes, which was suppressed by rapamycin or U0126, while any further PMA-mediated increase in synaptic AMPAR content was occluded in neurons overexpressing mTOR. (D) mTOR is a downstream effector of ERK in controlling protein synthesis. mTOR overexpression produced a significant increase in rates of protein synthesis as evidenced by the increase in AHA-incorporation rates. This mTOR mediated increase in protein synthesis rates was suppressed by the ERK blocker U0126.

SynGAP suppresses ERK activity [16], [39], however, it is not clear if ERK is the downstream mediator of SynGAP signaling that controls AMPAR levels. Moreover, although ERK is critical for increasing synaptic AMPARs in response to strong synaptic stimulation such as is used during LTP induction [40]–[43], its role in regulating synaptic AMPAR content under non-stimulated conditions has not been determined. To test the role of ERK in controlling basal synaptic strength, we measured mEPSC amplitudes following application of the ERK blocker U0126 or the PKC and ERK activator phorbol 12-myristate 13-acetate (PMA). Treatment with U0126 (25 µM, 6 hr) resulted in a non-significant trend decrease in mEPSC amplitudes, whereas PMA (1 µg/ml, 1 hr) significantly increased mEPSC amplitudes and pretreatment with U0126 for 30 min prior to PMA prevented the increase in mEPSC amplitudes (Fig. 2A). This increase in amplitudes was also recovered to control levels by treatment with the mTOR antagonist KU-0063794 (Fig. 2A). Application of PMA also strongly and consistently increased mEPSC frequency in these cells (mean ±SEM inter-event intervals, Control  = 4.87±0.92 s, n = 10; PMA = 0.50±0.11 s, n = 8; p<0.001). Interestingly, although pretreatment with U0126 rescued the post-synaptic change in mEPSC amplitudes, it did not recover the PMA-induced increase in mEPSC frequency (mean ±SEM inter-event intervals, Control  = 4.87±0.92 s, n = 10; U0126+PMA = 1.19±0.54 s, n = 11; p<0.005), suggesting an additional divergent mechanism of action, consistent with previous studies showing PMA increases neurotransmitter release from presynaptic terminals in a manner that may be independent of ERK and thus post-synaptic SynGAP activity [44], [45]. By carrying out FUNCAT technique treatment with PMA but not the inactive analog of PMA, P-2170 (4-alpha-Phorbol 12,13-Didecanoate) also enhanced protein synthesis (Fig. 2B). This increase was sensitive to treatment with either U0126 or KU-0063794 (Fig. 2B). Suppression of PMA's actions on mEPSC amplitudes and protein synthesis rates by KU-0063794 strengthens the evidence of a downstream role for mTOR. To test if ERK signaling regulates AMPAR incorporation and protein synthesis through mTOR, we first transfected neurons with mTOR. Overexpression of mTOR significantly increased mEPSC amplitudes in a manner sensitive to either rapamycin or U0126 (Fig. 2C). Moreover, the increase in mEPSC amplitude resulting from PMA application was occluded in mTOR overexpressing neurons, suggesting that mTOR acts downstream of ERK in regulating synaptic AMPAR levels (Fig. 2C). To directly measure protein synthesis rates we again conducted FUNCAT experiments under these conditions. Overexpression of mTOR enhanced protein synthesis rates and this was blocked by treatment with U0126 (Fig. 2D). Together these data indicate that ERK positively regulates levels of synaptic AMPARs through mTOR signaling and regulation of protein synthesis.

**Figure 3:**
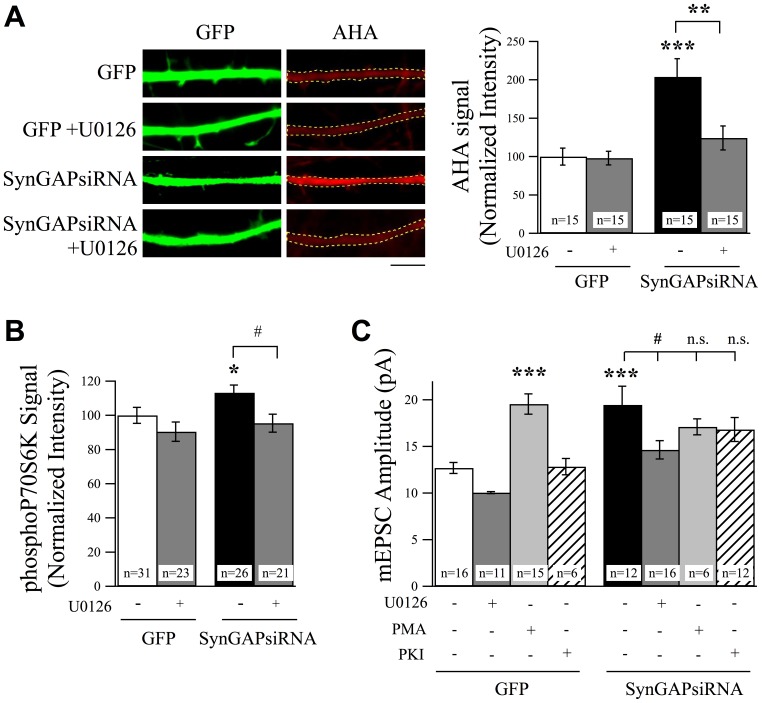
SynGAP Regulates ERK Signaling to Control Excitatory Synaptic Strength. (A) Example images and the normalized mean (+SEM) intensity of AHA signals are presented from experiments using FUNCAT to measure protein synthesis rates. SynGAPsiRNA-mediated increase in AHA-incorporation rates was suppressed by treatment with the ERK blocker U0126 (25 µM, 6 hr). Scale bar  = 5 µm. (B) Knockdown of SynGAP also increased levels of phosphorylated P70S6K, and this increase was suppressed by U0126. (C) U0126 rescued the increase in mEPSC amplitudes in neurons transfected with SynGAPsiRNA to control levels. The PMA-induced increase in mEPSC amplitudes in control neurons was occluded in neurons expressing SynGAPsiRNA. The increase in mEPSC amplitudes in cells expressing SynGAPsiRNA was not affected by the PKA blocker PKI 14–22 (1 µM, 6 hr). Note: the GFP control data in (C), shown for comparison, are also presented in Figure 2C.

Because removal of SynGAP enriched post-synaptic AMPAR content in a protein synthesis- and mTOR-dependent manner, we next tested whether or not this is mediated by ERK signaling. If this is the case, we predicted that the ERK blocker U0126 should rescue the increase in protein synthesis rates, as well as increased mEPSC amplitudes, in SynGAPsiRNA expressing neurons. Consistent with this hypothesis, treatment with U0126 (25 µM, 6 hr) returned protein synthesis rates to control levels in neurons expressing SynGAPsiRNA (Fig. 3A). Also levels of phosphorylated P70S6K, a major target for mTOR kinase activity, were enhanced in SynGAP knockdown cells and U0126 suppressed this increase to control levels, confirming that SynGAP normally limits protein synthesis under basal levels of activity by suppressing ERK signaling and limiting mTOR activation (Fig. 3B). To further confirm the ability of SynGAP to regulate synaptic AMPAR levels via ERK, we measured mEPSCs in SynGAP knockdown cells in the presence or absence of U0126 or PMA. In neurons expressing SynGAPsiRNA, U0126 significantly suppressed mEPSC amplitudes, whereas PMA did not produce any additive increase in mEPSC amplitudes (Fig. 3C). Because protein kinase A (PKA) is also involved in regulating AMPAR levels during development [46]–[48], we tested to see if PKA activity is suppressed by SynGAP. The cell permeable PKA inhibitor PKI 14–22 (1 µM, 6 hr) had no effect on mEPSC amplitude measurements in GFP expressing control neurons. Furthermore the increase in mEPSC amplitudes in SynGAPsiRNA expressing neurons was not affected by PKI, suggesting that PKA is not involved in basal, SynGAP-mediated AMPAR regulation under these experimental conditions (Fig. 3C). Taken together, these experiments indicate that SynGAP suppresses excitatory synaptic strength via ERK signaling and that SynGAP and mTOR utilize the same cellular signaling mechanisms to control synaptic strength.

### SynGAP Regulates Rheb to Limit Synaptic Strength

**Figure 4:**
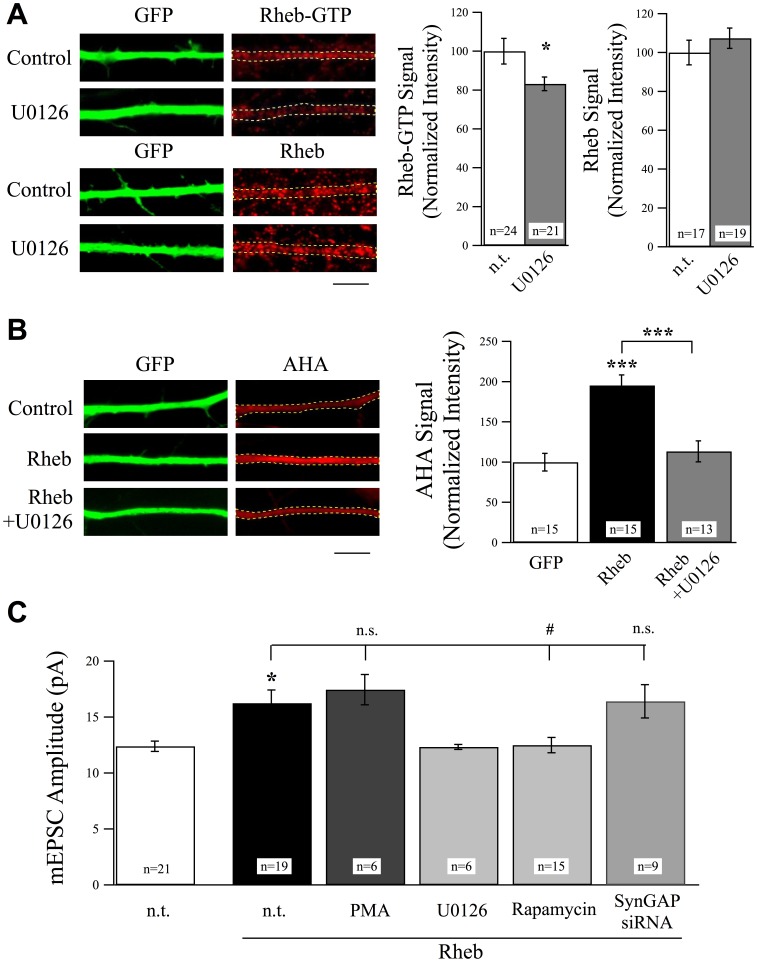
Rheb is a Downstream Effector of ERK and SynGAP Signaling to Regulate Excitatory Synaptic Strength. (A) Representative images of neurons stained with anti-Rheb-GTP antibody or total Rheb antibody in the presence or absence of the ERK blocker U0126 (25 µM, 6 hr). U0126 significantly suppressed levels of active Rheb without changing total Rheb levels. Scale bar  = 5 µm. (B) Example images and normalized AHA intensity values from experiments using FUNCAT to measure protein synthesis rates. Overexpression of Rheb in cortical neurons produced a significant increase in protein synthesis rates as evidenced by an increase in AHA-labeling rates. This increase in protein synthesis was suppressed by treatment with U0126 (25 µM, 6 hr). Scale bar  = 5 µm. (C) Overexpression of Rheb increased mEPSC amplitudes and was recovered by the treatment with U0126 (25 µM, 6 hr) or rapamycin (1 µM, 6 hr). Treatment with PMA (1 µg/ml, 6 hr) did not cause a further increase in mEPSC amplitudes in Rheb overexpressing neurons. Co-expression of Rheb did not produce a further increase in AMPAR-mEPSCs in neurons expressing SynGAPsiRNA.

To further elucidate the mechanisms by which SynGAP regulates excitatory synaptic strength, we examined the role of Rheb (ras homolog enriched in brain). Rheb is a small GTP-binding protein and an activator of mTOR [49]–[52]. Rheb may be indirectly activated by ERK, through inhibition of the Rheb suppressor TSC1/2 (tuberous sclerosis complex), which stimulates the hydrolysis of GTP-bound Rheb to GDP-bound Rheb [50], [51], [53]. If ERK and mTOR are downstream of SynGAP, as these data suggest, it raises the possibility that SynGAP may regulate synaptic strength via inhibition of Rheb. Furthermore, we wanted to know if Rheb affects synaptic strength by controlling translation under basal levels of synaptic activity, which has not previously been demonstrated. We first tested whether or not Rheb activity can be regulated by ERK. We conducted immunostaining analysis to measure the ratio of active Rheb to total Rheb using an anti-GTP-bound Rheb antibody and an anti-Rheb antibody in the presence or absence of the ERK blocker U0126. We found that U0126 significantly suppressed GTP-bound levels of Rheb without affecting total Rheb, confirming that Rheb can be regulated by ERK signaling (Fig. 4A).

We next determined whether or not Rheb regulates protein synthesis under conditions of basal levels of activity. Overexpression of Rheb increased protein synthesis above control levels and this increase could be suppressed by exposure to U0126 (Fig. 4B). To correlate translational activation with increased synaptic strength, we measured mEPSC amplitudes in neurons in which we overexpressed Rheb. Overexpression of Rheb increased mEPSC amplitudes, and this was also recovered to control levels by treatment with U0126 (Fig. 4C). Moreover, PMA, which significantly increased mEPSC amplitudes in non-transfected cells, did not produce further potentiation of AMPAR-mEPSCs in Rheb overexpressing neurons, providing evidence for the hypothesis that Rheb acts downstream of ERK signaling in controlling synaptic strength (Fig. 4C). To further confirm that Rheb and mTOR are coupled to regulate synaptic strength, we challenged neurons overexpressing Rheb with rapamycin. Treatment with rapamycin six hours prior to mEPSC recordings suppressed the increase in mEPSC amplitudes (Fig. 4C). These experiments demonstrate that ERK, Rheb, and mTOR signaling can increase protein synthesis leading to enhanced synaptic strength. In addition, they suggest that acute manipulation of this signaling pathway can rescue normal levels of synaptic strength.

If Rheb does indeed act as a mediator downstream of SynGAP to control AMPAR levels, we hypothesized that overexpression of Rheb will not produce an additive increase in mEPSC amplitudes in cells also expressing SynGAPsiRNA. Indeed, Rheb-mediated increase in mEPSC amplitudes was occluded in SynGAPsiRNA expressing neurons (Fig. 4C). Together, these data suggest that SynGAP regulates protein synthesis and synaptic strength in a manner dependent upon Rheb function.

### SynGAP is Required for Proper Homeostatic Synaptic Plasticity at Developing Cortical Synapses

**Figure 5:**
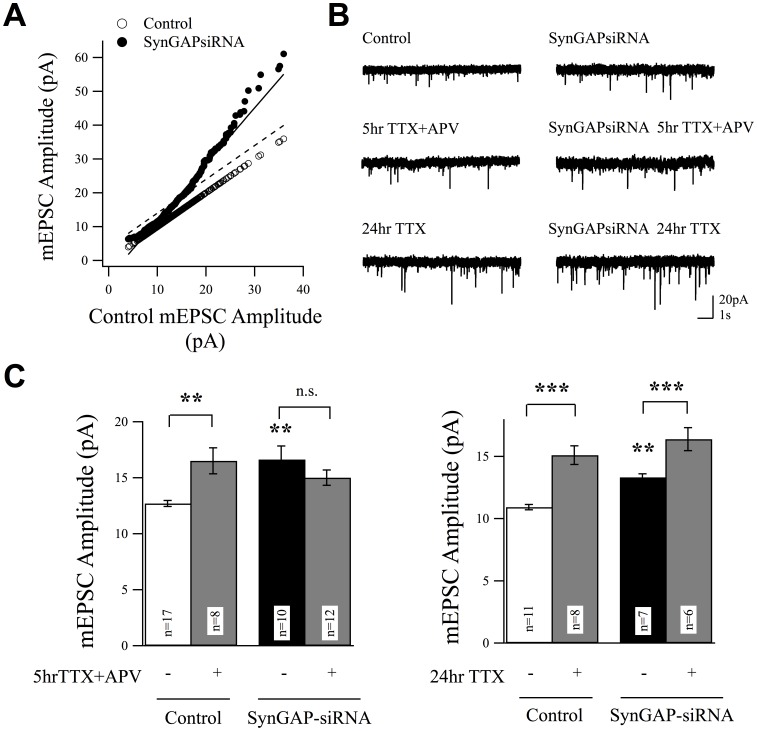
SynGAP is Required for Homeostatic Synaptic Scaling in Response to Acute, but Not Chronic, Neuronal Activity Blockade. (A) When ranked and plotted against control, non-treated cells (open circles), mEPSC event amplitudes measured in cells expressing SynGAPsiRNA (filled circles) exhibited a multiplicative relationship that can be fitted by the solid line (R^2^ = 0.98; p<0.001). The dashed line shows a predicted additive increase of amplitude values to the control events, for the sake of comparison. (B) Example traces of mEPSC recordings from cortical neurons after the indicated treatment (TTX: 2 µM; APV: 50 µM) in SynGAP-siRNA expressing neurons (right) or control neurons (left). (C) Acute activity blockade (5 hr TTX+APV) only scaled up mEPSC amplitudes in control neurons but not in neurons expressing SynGAPsiRNA. The ability to scale up mEPSC amplitudes in response to chronic activity blockade (24 hr TTX) was intact in both control and SynGAPsiRNA expressing neurons.

The increase in synaptic AMPAR levels in response to SynGAPsiRNA could be due to the addition of a constant number of AMPARs to each synapse (additive) or by scaling AMPAR content in a multiplicative manner. In order to examine whether the mEPSC event amplitudes in SynGAPsiRNA expressing neurons increased in an additive or multiplicative fashion, we ranked the amplitudes of all mEPSC events in each experimental condition. When we plotted these ranked mEPSC amplitudes we observed that the increase in mEPSC amplitudes in neurons expressing SynGAPsiRNA was multiplicative, relative to non-transfected controls, and could be fit by a linear function with a slope of 1.67 (R = 0.99, p<0.001) (Fig. 5A). A multiplicative increase in this distribution is characteristic of HSP [54]. HSP is a negative feedback mechanism that controls synaptic strength in a proportionate manner in response to changes in the amount of integrated synaptic input a neuron receives [23], [26], [27]. We have shown previously that cortical cultures at this age undergo strong bidirectional scaling of mEPSC amplitudes in response to changes in network activity. We therefore hypothesized that SynGAP might be involved in HSP. We tested the requirement for SynGAP in two forms of HSP: one that can be induced by chronic activity blockade (2 µM TTX for 24 hr) [54], and a second more rapid form that can be induced by acute activity blockade (2 µM TTX+50 µM APV for 5 hr) [25]. Scaling up of mEPSC amplitudes by chronic TTX requires transcriptional activation [26], [55], [56], whereas the more rapidly induced scaling does not require transcriptional activation but rather relies upon local protein synthesis in neuronal dendrites [21], [25], [57]. In control neurons both acute and chronic treatments significantly increased mEPSC amplitudes in control cells (Fig. 5B&C). However, in neurons expressing SynGAPsiRNA, the ability to respond to chronic activity blockade (24 hr TTX) was intact, whereas the scaling-up induced by acute activity blockade (5 hr TTX+APV) was prevented (Fig. 5B&C). Taken together, these results show that SynGAP is critical for proper translation-dependent scaling up of cortical synapses, consistent with a role in regulating protein synthesis.

### GluN2B and CaMKII Signaling Regulate SynGAP to Control Excitatory Synaptic Strength

**Figure 6:**
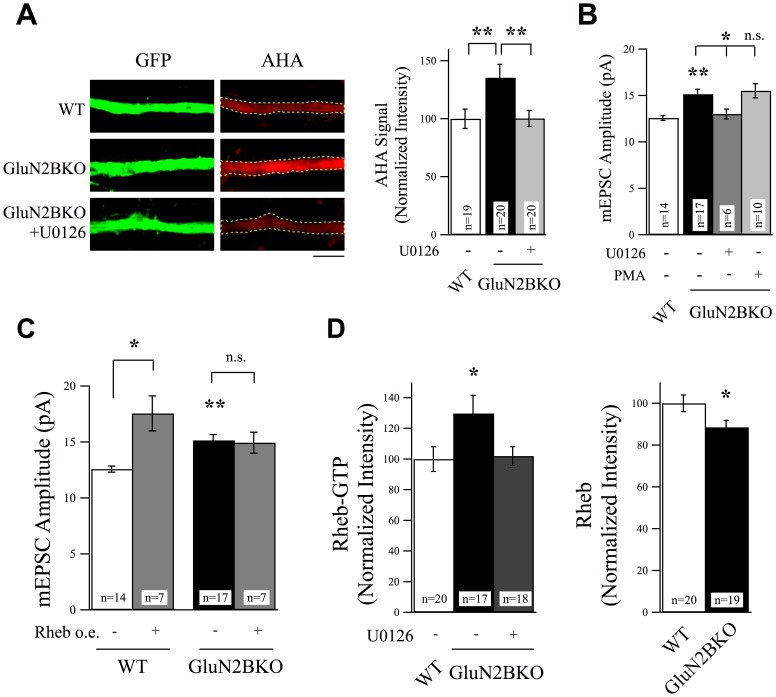
ERK and Rheb are Downstream of GluN2B Signaling. (A) GluN2B knockout neurons revealed a significant increase in protein synthesis rates as evidenced by measuring AHA incorporation rates. This increase was suppressed by treatment with U0126 (25 µM, 6 hr), scale bar  = 5 µm. (B) U0126 also suppressed the increase in mEPSC amplitudes observed in non-treated in GluN2B knockout neurons. Activation of ERK activity using PMA did not cause any further increase in mEPSC amplitudes in GluN2B knockout neurons. (C) The Rheb overexpression-induced increase in mEPSC amplitudes observed in WT neurons was occluded in GluN2B knockout neurons. (D) Rheb-GTP levels, determined using anti-Rheb-GTP antibody, increased in GluN2B knockout neurons. The total levels of Rheb signal were reduced in the absence of GluN2B.

SynGAP interacts preferentially with GluN2B-containing NMDARs [7], [58], which are also required for translation-dependent homeostatic synaptic plasticity [21]. Moreover, the phenotype of the SynGAP knockout animal is strikingly similar to the GluN2B knockout animal [7], [29], [59] and both SynGAP and GluN2B mutant animals display deficits in social interaction behaviors [13], [21]. Similar to removal of SynGAP, removal of GluN2B also increases synaptic AMPAR incorporation in a protein synthesis-dependent manner [21], [28], [60]. Together these data suggest that SynGAP acts as a direct downstream effector of GluN2B signaling, however there is little evidence for their ability to regulate the same downstream cellular mechanisms. To examine the links between GluN2B and SynGAP in regulating protein synthesis and synapse strength, we first investigated whether or not GluN2B also regulates ERK and Rheb activity. Genetic knockout of GluN2B significantly increased protein synthesis rates in a manner that could be recovered by treatment with U0126 (Fig. 6A). U0126 treatment also recovered mEPSC amplitudes in GluN2B knockout cells to control levels and activation of ERK signaling by PMA did not further potentiate AMPAR incorporation in GluN2B knockout neurons (Fig. 6B). These data suggest that ERK is regulated downstream of GluN2B signaling to control synaptic AMPAR levels, also in line with previous reports showing ERK is involved in GluN2B-dependent signaling [58], [61], however our data also show that this involves regulation of protein synthesis. Since Rheb acts downstream of SynGAP to set excitatory synaptic strength via regulation of protein synthesis, we suspected that Rheb would also be regulated downstream of GluN2B if SynGAP and GluN2B signaling are coupled. We therefore asked whether or not GluN2B controls synaptic AMPAR levels via Rheb. Indeed the increase in mEPSC amplitudes in Rheb overexpressing neurons was occluded in GluN2B null neurons (Fig. 6C). Furthermore, levels of GTP-bound Rheb were increased in GluN2B knockout neurons and could be suppressed by U0126 (Fig. 6D). Interestingly, levels of total Rheb protein were actually reduced in GluN2B knockout neurons, which could be the result of a compensatory mechanism to limit Rheb activity in the absence of GluN2B (Fig. 6D). Taken together, these data indicate that GluN2B-mediated signaling normally suppresses Rheb function, and the increase in AMPAR incorporation in GluN2B null neurons can be attributed, at least in part, to enhanced Rheb activation.

**Figure 7:**
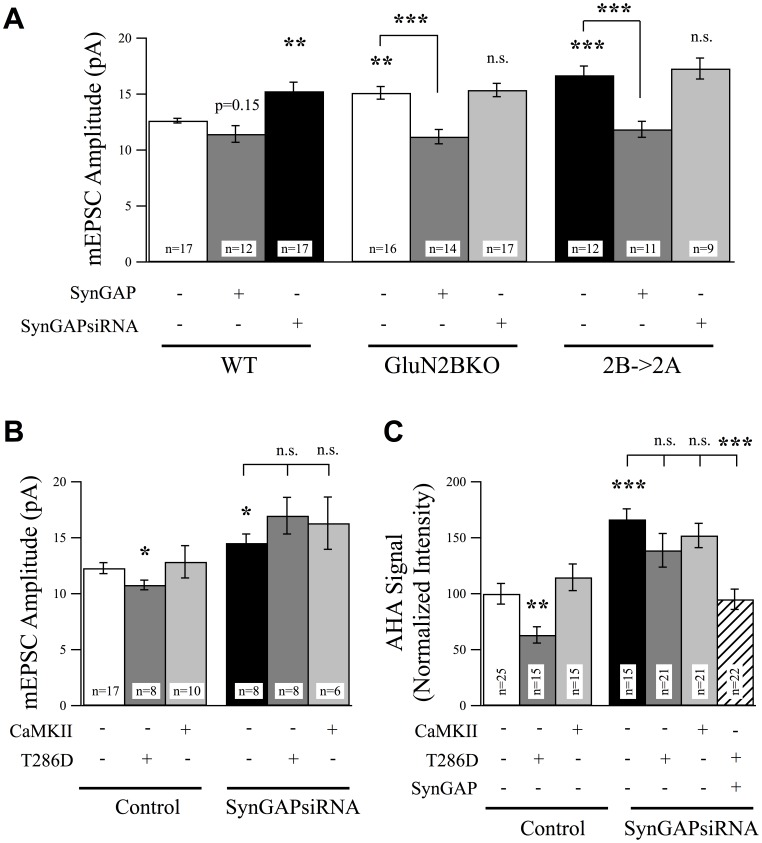
SynGAP Acts Downstream of GluN2B and CaMKII Signaling to Regulate Excitatory Synaptic Strength. (A) GluN2B knockout and 2B->2A replacement neurons exhibited a significant increase in mEPSC amplitudes, and this increase could be rescued by overexpression of SynGAP. However, any synGAPsiRNA-mediated increase in mEPSC amplitudes was occluded in both GluN2B knockout and 2B->2A neurons. (B) Overexpression of constitutively active alpha-CaMKII (T286D) significantly suppressed mEPSC amplitudes in control neurons but was unable to rescue the increase in mEPSC amplitudes in cells transfected with SynGAPsiRNA. (C) T286D overexpressing neurons exhibited suppressed protein synthesis rates as measured using FUNCAT. However, the increase in AHA-labeling rates in neurons expressing SynGAPsiRNA was not rescued by co-expression of T286D but could be recovered by co-expression of WT SynGAP.

To confirm that SynGAP is required for proper GluN2B signaling in regulating synaptic AMPAR content, and that this is unique to this NMDAR subunit, we conducted SynGAP loss-of-function and overexpression experiments in GluN2B knockout neurons and in neurons in which GluN2B has been genetically replaced with GluN2A (2B->2A). Using 2B->2A replacement neurons allows us to study the specific roles of GluN2B signaling, since NMDAR-mediated current is recovered by replacement with GluN2A in a GluN2B null background, ruling out effects resulting from general loss of NMDAR signaling [21]. AMPAR-mediated mEPSCs in both GluN2B knockout and 2B->2A neurons exhibited an increase in average amplitude (Fig. 7A), and this increase could be rescued by SynGAP overexpression in cells of either genotype (Fig. 7A). Furthermore, suppressing SynGAP expression did not further increase mEPSC amplitudes in GluN2B knockout nor 2B->2A neurons, predicting that GluN2B acts upstream of SynGAP (Fig. 7A).

How do GluN2B-containing NMDARs regulate SynGAP function to suppress synaptic strength? We tested the potential involvement of CaMKII for several reasons; CaMKII preferentially interacts with GluN2B over GluN2A [17], [18], [62], [63], SynGAP is activated and phosphorylated by CaMKII [9], and SynGAP, CaMKII, and NMDARs are associated in a complex that can regulate ERK activity [30], [58]. In addition, CaMKII is an effector of GluN2B signaling that can suppress synaptic AMPAR incorporation under basal levels of activity [21]. Consistent with a role for CaMKII in basal regulation of synaptic strength, overexpression of a constitutively active version of CaMKII (T286D), but not WT CaMKII, was sufficient to significantly reduce mEPSC amplitudes in cortical neurons (Fig. 7B). If CaMKII is signaling upstream of SynGAP, we hypothesized that c.a. CaMKII would not be able to suppress AMPAR-mEPSC amplitudes in the absence of SynGAP. Supporting this idea, we observed that the increase in mEPSC amplitudes in SynGAPsiRNA expressing neurons was not affected by co-expression of T286D (Fig. 7B). Additionally, we measured protein synthesis and found that rates of protein synthesis could be reduced in cells overexpressing CaMKII-T286D but not WT CaMKII, and increased protein synthesis in SynGAPsiRNA expressing cells was not significantly reduced by either version of CaMKII in the absence of SynGAP (Fig. 7C). Finally, to confirm that CaMKII normally acts under conditions of basal activity to limit synaptic strength, we tested the role of CaMKII in protein synthesis-dependent HSP. Treatment with TTX and the CaMKII inhibitor KN93 significantly increased mEPSC amplitudes mimicking the actions of TTX+APV (Fig. 7D; c.f. Fig. 5). However, application of TTX and KN93 did not further increase mEPSC amplitudes in neurons expressing SynGAPsiRNA (Fig. 7D). This suggests that GluN2B and CaMKII are upstream of SynGAP signaling and this signaling complex plays a critical role in limiting synaptic AMPAR content at developing cortical synapses. Furthermore, our data demonstrate a role for this signaling cascade in HSP.

## Discussion

**Figure 8:**
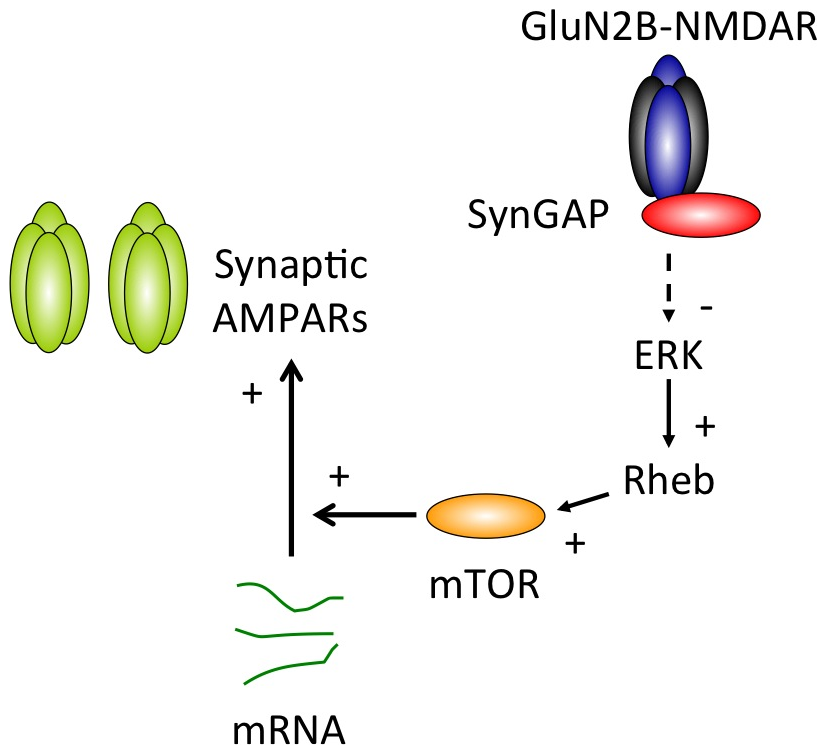
Proposed Model of the SynGAP-Mediated Cellular Signaling Pathway that Regulates Translation and Synaptic AMPAR Content. Our data predict the following model: under conditions of basal neuronal activity, association of GluN2B and CaMKII links SynGAP to suppression of ERK signaling. ERK can activate Rheb, which, in turn, activates mTOR. Through suppression of mTOR function this mechanism inhibits protein synthesis and limits AMPAR contribution at developing cortical networks. Our data show that this signaling pathway is critical for developmental synaptic plasticity as translation-dependent homeostatic synaptic plasticity is disrupted in the absence of GluN2B or SynGAP.

In order to control the strength of excitatory synaptic transmission and maintain proper E/I balance in developing brain circuits, levels of synaptic AMPARs are tightly regulated. In this study, we report that SynGAP indeed acts as a critical regulator of AMPAR content at developing cortical synapses, in part, by limiting protein synthesis. Based upon our findings we propose a mechanistic model whereby SynGAP suppresses ERK signaling, which normally promotes Rheb function and mTOR activation (Figure 8). We also show that this cellular signaling cascade is active under basal levels of synaptic activity and is tightly associated with GluN2B-mediated NMDARs and CaMKII. Furthermore, we demonstrate that this signaling cascade is required for proper induction of translation-dependent homeostatic synaptic plasticity.

We found here that removal of SynGAP from cortical neurons increases ERK activity and leads to an increase in the strength of excitatory synapses, which is consistent with previous reports [15], [16], [39]. ERK is an important cellular kinase that senses a variety of extracellular signals to modulate neuronal function. ERK is important for regulating synaptic AMPAR incorporation during LTP induction [40]–[43], [64]. However, our data reveal that SynGAP also suppresses ERK activity and limits synaptic AMPARs, under basal conditions of synaptic activity and that this normally suppresses incorporation of calcium permeable AMPARs, due to the increase in NASPM sensitive AMPAR current in SynGAP siRNA expressing neurons. This is consistent with previous reports showing that GluR1 expression is regulated by local protein synthesis [65] and suppression of basal NMDAR signaling increases the expression of calcium permeable receptors [25]. It will be interesting in future experiments to explore the possibility that developmental changes in expression of calcium permeable AMPARs in cortex is regulated by SynGAP [66]. Our data also show that SynGAP signaling limits protein synthesis by repressing mTOR function through suppression of ERK. Based upon previous studies we predict that activation of ERK may lead to increased mTOR function through loss of inhibition of the TSC 1/2 complex, however this remains to be directly tested in cortical neurons [50], [51], [67]. Together, these results link SynGAP function to mTOR activation and protein synthesis, in addition to showing that ERK signaling plays an important role in setting levels of synaptic AMPARs under non-stimulated conditions.

GluN2B-containing NMDARs can act to suppress excitatory synapse strength. Genetic removal, or suppressed expression, of these receptors in developing networks causes increased AMPAR signaling [21], [28], [60], [68], [69]. SynGAP is one of the most abundant proteins at the PSD and associates with NMDARs through direct interaction with PSD scaffold proteins [7], [8]. Moreover, ERK activity can be regulated by both GluN2B and SynGAP [16], [58] and SynGAP is known to associate preferentially with GluN2B-containing NMDARs [58]. Here we demonstrate that SynGAP indeed acts in a subunit specific manner playing a critical role downstream of GluN2B to suppress AMPAR current. Our experiments used genetic manipulation, including GluN2B knockout and replacement of GluN2B by GluN2A, in order to avoid problems associated with assessing NMDAR function using subunit selective antagonists. Together our data indicate that SynGAP is a major effector of GluN2B function and provide an explanation for the phenotypic similarities that exist between global SynGAP and GluN2B subunit genetic knockout animals [15], [29], [39], [59].

SynGAP is phosphorylated and activated by CaMKII [9]. Furthermore, SynGAP, CaMKII, and the NMDAR complex are all closely associated at the PSD [30]. CaMKII is a known cellular effector of NMDAR function and although its importance in LTP is well established previous studies have also shown that overexpression of constitutively active CaMKII (T286D) reduces both spontaneous and evoked synaptic AMPAR-mediated currents [21], [70]. Here we show that overexpression of CaMKII-T286D in cortical neurons reduces protein synthesis rates, thus providing a potential mechanism to explain how excitatory synaptic function is negatively regulated downstream of GluN2B and CaMKII. These studies therefore provide a molecular link between GluN2B-containing NMDARs, and regulation of protein synthesis through their association with CaMKII and SynGAP.

While both SynGAP and NMDARs containing the GluN2B subunit have been shown to be required during LTP induction [39], [59], [71]–[73], they also suppress AMPAR incorporation under basal levels of activity [14]–[16], [21], [28], [39], [59], [60], [68]. Strong expression of GluN2B at developing cortical synapses coincides with a period of low AMPAR to NMDAR current ratios. Our data suggest that this could be maintained, in part, by suppression of protein synthesis by GluN2B via SynGAP. In addition, GluN2B has been shown to be critically involved in protein synthesis dependent homeostatic synaptic plasticity (HSP). HSP, also known as synaptic scaling, provides a negative feedback mechanism that can increase excitatory synaptic strength in response to decreases in overall levels of synaptic activity. This synaptic scaling occurs in a multiplicative manner. Here we show that suppression of SynGAP both mimics and occludes the ability of neurons to scale up synaptic strength in response to acute manipulation of activity therefore providing strong evidence that SynGAP associated signaling is involved in a rapidly induced, protein synthesis dependent form of synaptic scaling.

While our SynGAP and GluN2B loss of function experiments implicate these proteins in HSP, it is unclear how they can be required for both the increase in AMPAR signaling seen in LTP as well as limiting AMPAR under non-stimulated conditions. We suggest this could be due to a decoupling of the association of these proteins in response to strong synaptic activation. In support of this idea, SynGAP is associated with, and phosphorylated by, CaMKII under basal levels of activity [30]. However, strong activation of NMDARs results in dissociation of SynGAP from CaMKII and drives dephosphorylation of SynGAP [30]. Moreover, SynGAP moves out of the PSD upon strong NMDAR activation [74]. We therefore propose that SynGAP is responsible for limiting incorporation of synaptic AMPARs, under basal levels of activity, but that this signaling pathway is decoupled following strong NMDAR activation allowing potentiation of synaptic strength, for example during LTP. It will be critical in future experiments to further detail the spatial redistribution and levels of SynGAP activity following HSP and LTP induction.

Interestingly, increased synthesis of synaptic proteins is observed in several animal models of autism [5], [6], [75] and exome sequencing of autistic patients has identified several *de novo* mutations in genes involved in regulation of protein synthesis [76], [77]. In neurons mTOR is known to be involved in regulation of protein synthesis and synaptic transmission [36], [78]–[82] and dysregulation of mTOR activity has been implicated in ASD [35], [83], [84]. In fact genetic removal of P70S6 kinase, the primary substrate of mTOR, or suppression of mTOR-mediated translation, can rescue phenotypic alterations in mouse models of ASD [5], [34], [38]. Consistent with these observations SynGAP mutant mice have recently been shown to display autistic-like phenotypes [14]. Our studies provide a molecular mechanism to explain how genetic suppression of SynGAP could enhance protein synthesis in an mTOR-dependent manner, and therefore link SynGAP dysfunction with ASD [14]. Importantly, our results demonstrate that SynGAP is a major effector downstream of GluN2B. Interestingly, mice with selective cortical deletion of GluN2B also display autistic-like behaviors [21] and exome sequencing shows that mutations in GluN2B are highly associated with non-syndromic ASD [77].

Finally, fragile X syndrome (FXS) is the most common form of inherited intellectual disability as well as a leading cause of autism [85], [86]. FXS is caused by expanded CGG repeats in the promoter region of the fragile X mental retardation 1 gene (*Fmr1*), leading to hypermethylation of *Fmr1* and silencing of the gene [87]. FMRP is present in dendrites and is an mRNA binding protein that inhibits translation of specific mRNAs. Exaggerated protein synthesis is observed in FMRP knockout mice [88]. Notably, mTOR signaling is also enhanced in FMR1 knockout mice [35] and ERK signaling has been shown to be hypersensitive to regulation of elevated protein synthesis rates in the absence of FMR1 [89]. Moreover, genetic removal of p70S6K can correct the molecular, synaptic, and behavioral phenotypes observed in FMR1 knockout mice [34]. Because our results also demonstrate that mTOR and ERK signaling are potentiated in the absence of SynGAP, it is highly plausible that there might be a crosstalk between SynGAP and FMRP. Future experiments will be required to address these questions.

In conclusion, our results provide a mechanistic link in our understanding of how SynGAP regulates excitatory synaptic strength and how SynGAP mutations could lead to brain disorders such as non-syndromic ID and ASD through its ability to regulate protein synthesis.
